# Pontin/Tip49 negatively regulates JNK-mediated cell death in *Drosophila*

**DOI:** 10.1038/s41420-018-0074-1

**Published:** 2018-07-09

**Authors:** Xingjun Wang, Xirui Huang, Chenxi Wu, Lei Xue

**Affiliations:** 1 Institute of Intervention Vessel, Shanghai 10th People’s Hospital, Shanghai Key Laboratory of Signaling and Diseases Research, School of Life Science and Technology, Tongji University, 1239 Siping Road, Shanghai, 200092 China; 2 Department of Neuroscience, Scripps Research Institute, Florida, 130 Scripps Way, Jupiter, FL USA; 3 College of Chinese Medicine, North China University of Science and Technology, 21 Bohai Road, Tangshan, 063210 China

**Keywords:** Genetics, Developmental biology

## Abstract

Pontin (Pont), also known as Tip49, encodes a member of the AAA+ (*A*TPases *A*ssociated with Diverse Cellular *A*ctivities) superfamily and plays pivotal roles in cell proliferation and growth, yet its function in cell death has remained poorly understood. Here we performed a genetic screen for dominant modifiers of Eiger-induced JNK-dependent cell death in *Drosophila*, and identified Pont as a negative regulator of JNK-mediated cell death. In addition, loss of function of Pont is sufficient to induce cell death and activate the transcription of JNK target gene *puc*. Furthermore, the epistasis analysis indicates that Pont acts downstream of Hep. Finally, we found that Pont is also required for JNK-mediated thorax development and acts as a negative regulator of JNK phosphorylation. Together, our data suggest that *pont* encodes a negative component of Egr/JNK signaling pathway in *Drosophila* through negatively regulating JNK phosphorylation, which provides a novel role of ATPase in Egr-JNK signaling.

## Introduction

Pontin (Pont), also known as Tip49, Tip49a, NMP238, TAP54α, Ruvbl1, Rvb1, pontin52^1–5^, belongs to the superfamily of AAA+ ATPases (ATPases Associated with Diverse Cellular Activities), which is the extension of the known AAA family^6,7^. Pont family proteins are evolutionally conserved from yeast to humans^8^, and have been reported to play vital roles in regulating gene transcription^9,10^, cell proliferation^11,12^ and growth^13–15^. However, the role of Pont in regulating cell death in development has remained elusive.

The c-Jun N-terminal kinase (JNK) pathway is evolutionarily conserved from fruit flies to humans, and plays diverse biological functions including stress response, cell death, proliferation, tumor metastasis, longevity and sleep control^16–25^. JNK pathway is also involved in neurodegenerative diseases such as Parkinson’s disease^26,27^ and Alzheimer’s disease^28–30^. In *Drosophila*, the tumor necrosis factor ortholog Eiger (Egr) binds to its receptors Wengen^31^ or Grindelwald^32^ to activate the conserved dTAK1 (JNKKK)–Hep (JNKK)–Bsk (JNK) kinase cascade^33,34^, which triggers cell death through downstream transcription factors like AP1 and FoxO^35–37^. We have previously performed a genetic screen for dominant modifiers of Egr-induced cell death, and have identified additional factors that regulate JNK-mediated cell death^17,38–43^.

In this report, we characterized the ATPase Pont as a negative regulator of Egr-JNK signaling in *Drosophila*. We found that loss of function of *pont* enhances Egr-induced JNK-mediated cell death, while gain of function of *pont* suppresses it. Furthermore, we showed that loss of function of *pont* activated JNK target gene *puc* transcription and Pontin acted downstream of Hep in the Egr-JNK pathway. Third, we found that Pontin was required for the growth of the scutellum in the developing thorax. Finally, we demonstrated that loss of function of *pont* was sufficient to elevate the phosphorylation of JNK in vivo. Collectively, our genetic work clarifies a role of ATPase Pontin in regulating Egr-JNK signaling during the development of the *Drosophila*.

## Results and discussion

### Loss of function of *pont* promotes Egr-induced cell death in *Drosophila*

**Fig. 1:**
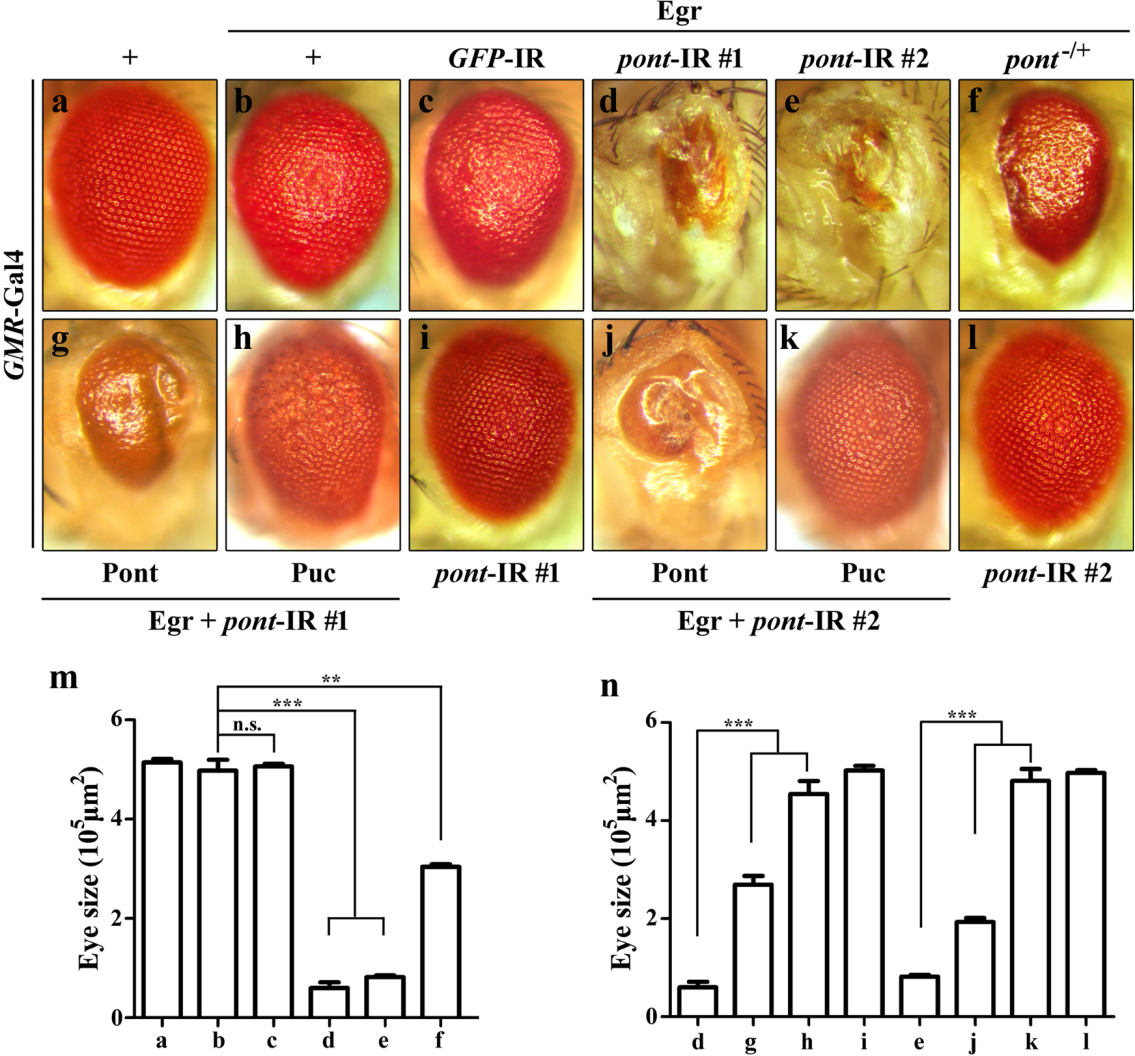
Loss of function of Pont promotes Egr-induced eye phenotype. Light images of adult eyes are shown (**a**–**l**). *GMR*-Gal4 was used as a control (**a**), or to drive the expression of Egr (**b-h**, **j**, **k**) or *pont*-IR #1 (**d**, **g**, **h**, **i**), *pont*-IR #2 (**e**, **j**, **k**, **l**), *GFP*-IR #1 (**c**). *GMR*>Egr-induced rough eye phenotype (**b**) was enhanced by expression of *pont*-IR (**d**, **e**) or by *pont* heterozygous mutant (**f**) and not by expression of a random *GFP*-IR (**c**). *pont*-IR-enhancing Egr-induced eye phenotype was suppressed by expression of Pont (**d**, **e**, **g**, **j**, **n**) or Puc (**d**, **e**, **h**, **k**, **n**). Expression of *pont*-IR alone produced no evident eye phenotype (**i**, **l**, **n**). **m** Statistical analysis of the eye size in (**a**–**f**). Error bars means ± SEM, ***P ≤ 0.001, **P ≤ 0.01, n.s. not significant. **n** Statistical analysis of the eye size of the indicated genotypes. Error bars means ± SEM, ***P ≤ 0.001. Genotypes: *GMR*-Gal4/+ (**a**); *UAS*-Egr^W^/+; *GMR*-Gal4/+ (**b**); *UAS-*Egr^W^/ *UAS*-*GFP*-IR*; GMR*-Gal4/+ (**c**); *UAS-*Egr^W^/+; *GMR*-Gal4/*UAS*-*pont*-IR#1 (**d**); *UAS-*Egr^W^/ *UAS*-*pont*-IR #2*; GMR*-Gal4/+ (**e**); *UAS-*Egr^W^/+; GMR-Gal4/ *pont*^5.1^ (**f**); *UAS-*Egr^W^/*UAS*-Pont*; GMR*-Gal4/*UAS*-*pont*-IR #1 (**g**); *UAS-*Egr^W^/+; *GMR*-Gal4/*UAS*-*pont*-IR #1 *UAS*-Puc (**h**); *GMR*-Gal4/*UAS*-*pont*-IR #1 (**i**); *UAS-*Egr^W^/ *UAS*-*pont*-IR #2*; GMR*-Gal4/*UAS*-Pont (**j**); *UAS-*Egr^W^/ *UAS*-*pont*-IR #2*; GMR*-Gal4/*UAS*-Puc (**k**); *UAS*-*pont*-IR #2/+; GMR-Gal4/*+*(**l**)

Ectopic expression of Egr in the developing eye driven by *GMR*-Gal4 triggers JNK-mediated cell death^38,39^ and produces dosage-dependent eye phenotypes—a rough eye from weak Egr expression (*GMR*>Egr^W^, Fig. 1b–m) and a small eye from strong Egr expression (*GMR*>Egr^S^, Fig. 5b–i). We have performed a genetic screen for dominant modifiers of the *GMR*>Egr phenotypes, and have identified additional factors that modulate Egr-induced JNK-mediated cell death^40,42^.

Pont was identified as a suppressor from the screen as knockdown of *pont* dramatically enhanced *GMR*>Egr^W^-induced eye phenotype, producing rather small eyes (Fig. 1d–m), reminiscent of strong Egr expression in the eye (Fig. 5b)^17^. *pont* encodes a member of the AAA+ family of helicases (*A*TPases *A*ssociated with Diverse *A*ctivities)^6,7^. To further examine the role of *pont* in Egr-induced cell death, we used a second *pont*-IR (Fig. S1), as expected, which also exacerbated Egr-induced cell death in the eye (Fig. 1e–m), while expression of *pont*-IR alone failed to produce any obvious phenotype in the eye (Fig.1i, l, n). Consistently, when removing one copy of *pont*^11^, *GMR*>Egr-induced rough eye phenotype was also significantly enhanced (Fig. 1f–m), while deleting one copy of endogenous *pont* alone had no obvious phenotype in the eye (data not shown). To exclude the possible competition for Gal4 protein by the *UAS* lines, *GFP*-IR was adopted. Expression of *GFP*-IR failed to duplicate *pont*-IR effect in Egr-induced eye phenotype (Fig. 1c–m). Furthermore, loss of *pont* enhancing Egr-induced cell death phenotype was restored by the overexpression of Pont (Fig. S2) in the eye (Fig. 1d, e, g, j, n). Collectively, the data indicate loss of function of *pont* function promotes Egr-induced cell death in the developing eye and *pont* acts as a negative regulator of JNK signaling pathway.

### Loss of function of *pont* triggers JNK-mediated cell death

**Fig. 2:**
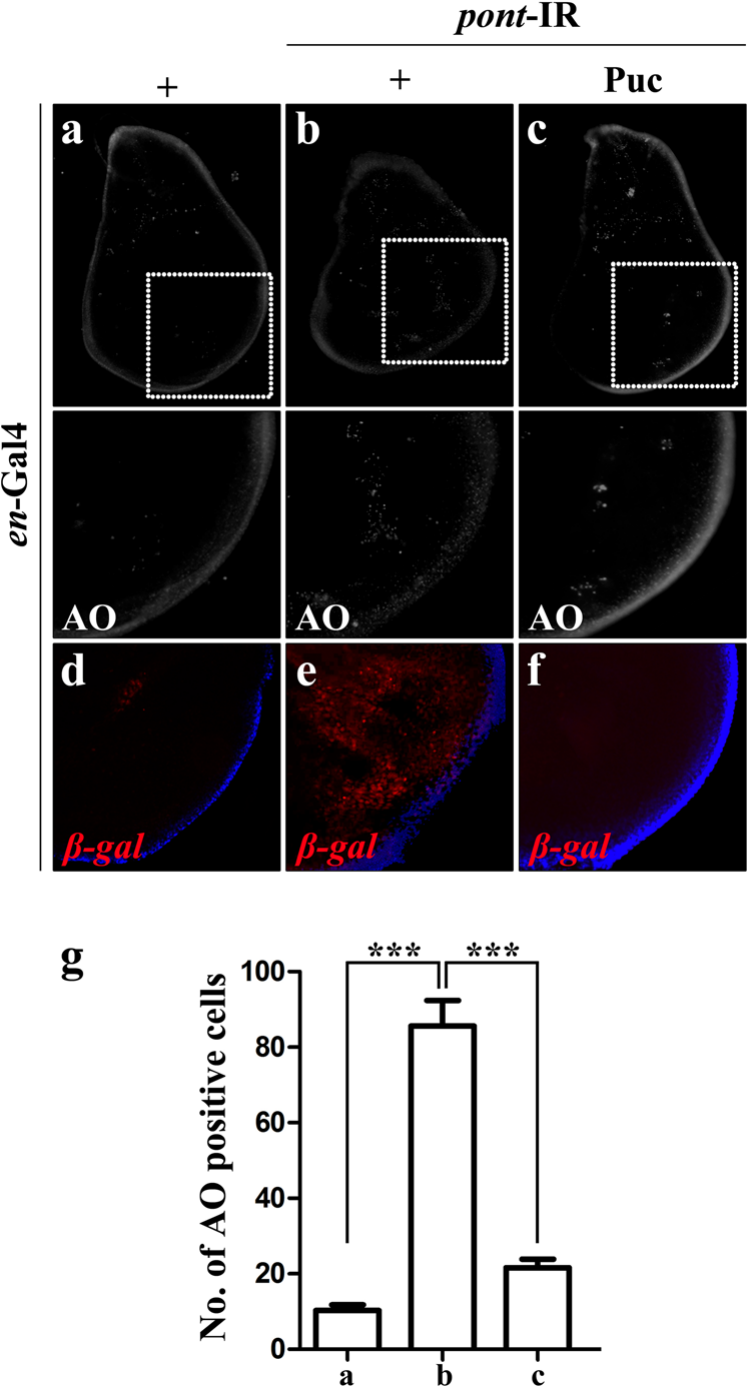
Loss of function of Pont triggers JNK-mediated cell death in the larval wing discs. Fluorescent images of acridine orange staining of the third-instar larva wing disc (**a**–**c**) and β-galactosidase immunostaining for *puc*-LacZ (**d**–**f**) are shown. Knockdown of *pont* in the posterior compartment of the wing disc driven by *en*-Gal4 (**a**, **d**) triggered cell death (**b**) and *puc* activation (**e**), which were suppressed by expressing of Puc (**c**, **f**). The lower panels are the magnification of the boxed area in the upper panels. **g** Statistical analysis of acridine orange-positive cells in (**a**–**c**). Error bars means ± SEM, ***P ≤ 0.001. Scale bar for **a**–**c**, 200 μm. Scale bar for **d**–**f**, 100 μm. Genotypes *en*-Gal4/+ (**a**); *en*-Gal4/*UAS*-*pont*-IR #2 (**b**); *en*-Gal4 *UAS*-*pont*-IR #2/+; *UAS*-Puc/+ (**c**); *en*-Gal4/+; *puc*^E69^/+ (**d**); *en*-Gal4/*UAS*-*pont*-IR #2; *puc*^E69^/+ (**e**); *en*-Gal4 *UAS*-*pont*-IR #2/+; *puc*^E69^/*UAS*-Puc (**f**)

We further characterized the role of Pont in regulating cell death and Egr-JNK signaling activation. RNA interference (RNAi)-mediated knockdown of Pont by *en*-Gal4 in the developing wing disc provoked strong cell death in the wing discs (Fig. 2b–g) compared with the control (Fig. 2a–g). The same results were obtained by expressing of *pont*-IR in the wing pouch driven by *sd*-Gal4 (Fig. S3a, b, c and g). To examine if loss of function of *pont*-induced cell death was due to the activation of caspase signaling, we checked the immunostaining of cleaved caspase-3 in the developing wing disc. Though expression of *pont*-IR triggered strong cell death in the wing disc (Fig. S3a, b, c and g), it failed to activate caspase signaling (Fig S4a, b and c). Taken together, the data indicate *pont* is required for the regulation of cell death and loss of function of *pont* triggers caspase-independent cell death. To investigate the physiological role of *pont* in JNK activation, we examined the expression pattern of *puckered (puc)* in *pont* loss-of-function background. *puc* encodes a JNK phosphatase whose expression is positively regulated by the Egr-JNK pathway^17,44,45^. Here, we used the *puc*-LacZ expression of the *puc*^*E69*^ enhancer-trap allele as a readout of the JNK activity in vivo^46^. The *en*>*pont*-IR strongly activated *puc* transcription in the posterior compartment of the wing compared with *en*-Gal4 (Fig. 2d, e). As a positive control, expression of Hemipterous (Hep), a JNK kinase^33,34,47^, also triggered *puc* activation in the wing disc (Fig. S5a and b). The same results were also obtained in *sd*>*pont*-IR, which activated *puc* transcription in the whole wing pouch (Fig. S3d, e and f). Collectively, these data suggest that endogenous *pont* is required for regulating the transcription of *puc*. To further clarify the role of Pont in regulating JNK signaling, we checked if loss of JNK signaling could abolish *pont*-IR induced cell death and *puc* activation. As expected, *pont*-IR induced cell death and *puc* activation were significantly suppressed by expression of a JNK phosphatase Puc^17,44,45^ (Fig. 2c, f, g). Consistently, *pont*-IR enhancing *GMR*>Egr-induced small eye phenotype was also restored by expression of Puc (Fig. 1d, e, h, k, n). Collectively, the data indicate loss of function of *pont* induced JNK activation and initiated JNK-mediated cell death.

### Loss of function of *pont* produces JNK-mediated cell death phenotype in adults

**Fig. 3:**
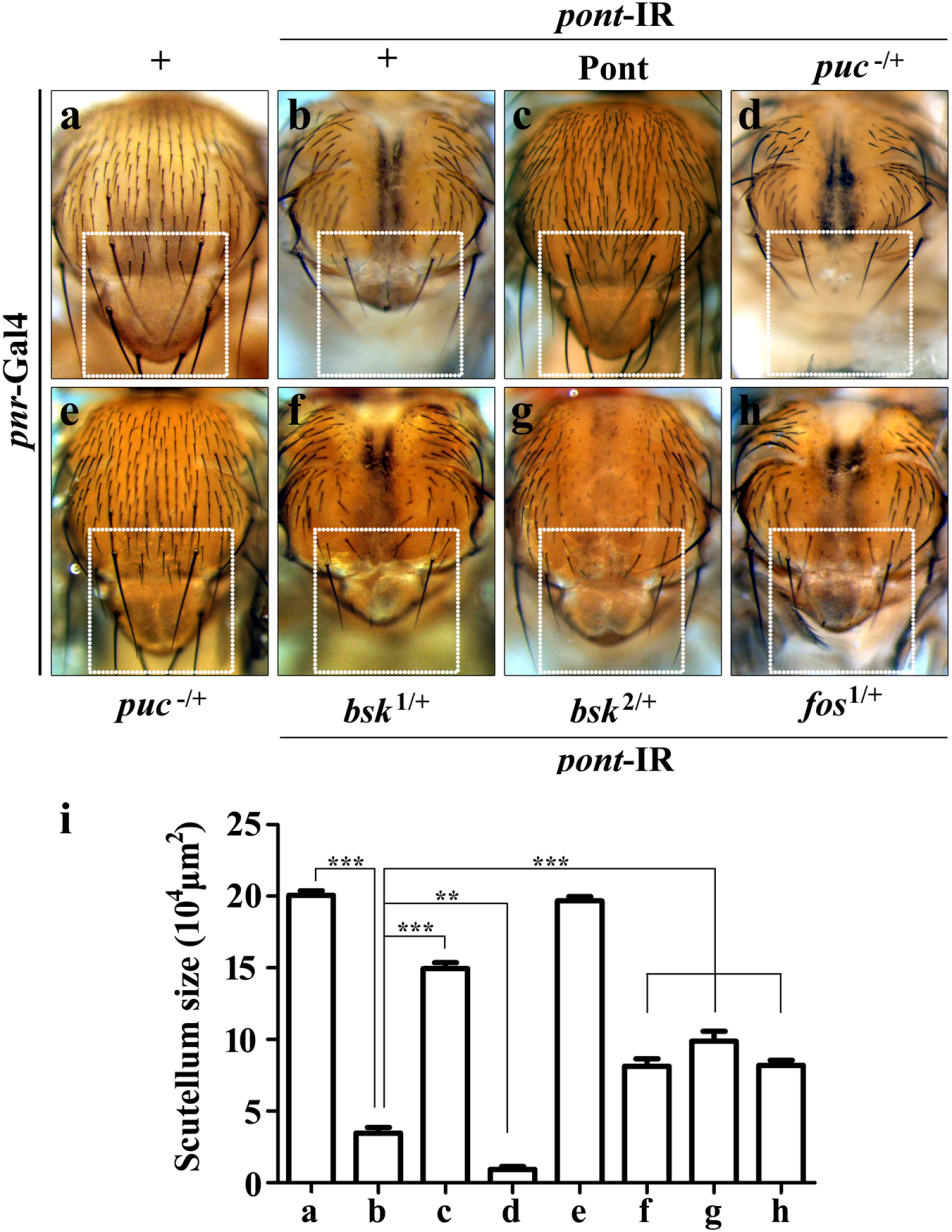
Loss of function of Pont produces JNK-mediated cell death phenotype in adults. Light images of *Drosophila* thoraxes (**a**–**h**) are shown. *pnr*-Gal4 was used as a control (**a**), or to drive the expression of *pont*-IR (**b**–**d**, **f**–**h**) or *UAS-*Pont (**c**). *pnr*>*pont*-IR induced small scutellum phenotype (**b**, **i**), was suppressed by expression of Pont (**c**, **i**) and enhanced in *puc* heterozygous mutant background (**d**, **i**), while *puc* heterozygous mutant alone showed no effect on the scutellum (**e**, **i**). *pnr*>*pont*-IR-induced small scutellum phenotype (**b**, **i**) was suppressed by in *bsk* heterozygous mutant (**f**, **g**, **i**) and *fos* heterozygous mutant (**h**, **i**) background. **i** Statistical analysis of the scutellum size in (**a**–**h**). Error bars means ± SEM, ***P ≤ 0.001, **P ≤ 0.01. Genotypes: *pnr*-Gal4/+ (**a**); *UAS*-*pont*-IR #2/+; *pnr*-Gal4/+ (**b**); *UAS*-*pont*-IR #2/*UAS*-Pont; *pnr*-Gal4/ *UAS*-Pont (**c**); *UAS*-*pont*-IR #2/+; *pnr*-Gal4/*puc*^E69^ (**d**); *pnr*-Gal4/*puc*^E69^ (**e**); *UAS*-*pont*-IR #2/+; *pnr*-Gal4/*bsk*^1/+^(**f**); *UAS*-*pont*-IR #2/+; *pnr*-Gal4/*bsk*^2/+^(**g**); *UAS*-*pont*-IR #2/+; *pnr*-Gal4/*fos*^1/+^(**h**)

Targeted knockdown of endogenous *pont* function in the thorax driven by *pannier*-Gal4 (*pnr*-Gal4) induced strong cell death and generated a reduced scutellum phenotype (Fig. 3a, b, i), mimicking Egr and Hep activation (Figs. 6b and 7g)^17,40^. Furthermore, *pnr*>*pont*-IR induced small scutellum phenotype was fully reverted by co-expressing Pont (Fig. 3b, c, i). The data indicate Pont is required for the regulation of the thorax development. To investigate the exact relationship between Pont and JNK signaling, we checked if *pnr*>*pont*-IR-induced defect was dependent on JNK pathway. As expected, *pnr*>*pont*-IR-induced developing defect in the scutellum was significantly exacerbated in the *puc* heterozygous mutant background (Fig. 3b, d, i), while depleting one copy of *puc* showed no evident phenotype in the the thorax (Fig. 3e–i). Reducing JNK signaling by removing one copy of endogenous *bsk*^48^ moderately suppressed *pnr*>*pont*-IR-induced defects in the notum (Fig. 3b, f, i). The same suppression effect was observed in another *bsk* mutant background^48^ (Fig. 3b, g, i). Upon stress, JNK translocates into the nucleus to phosphorylate the transcription factor Fos, and thus regulates cell death, tumor invasion and dorsal closure^35,37,49,50^. We found that *fos* heterozygous mutant compromised *pnr*>*pont*-IR-induced small scutellum phenotype (Fig. 3b, h, i). Based on the above data, we conclude that Pont regulates thorax development through JNK-Fos signaling.

### Loss of function of *pont* triggers JNK phosphorylation

**Fig. 4:**
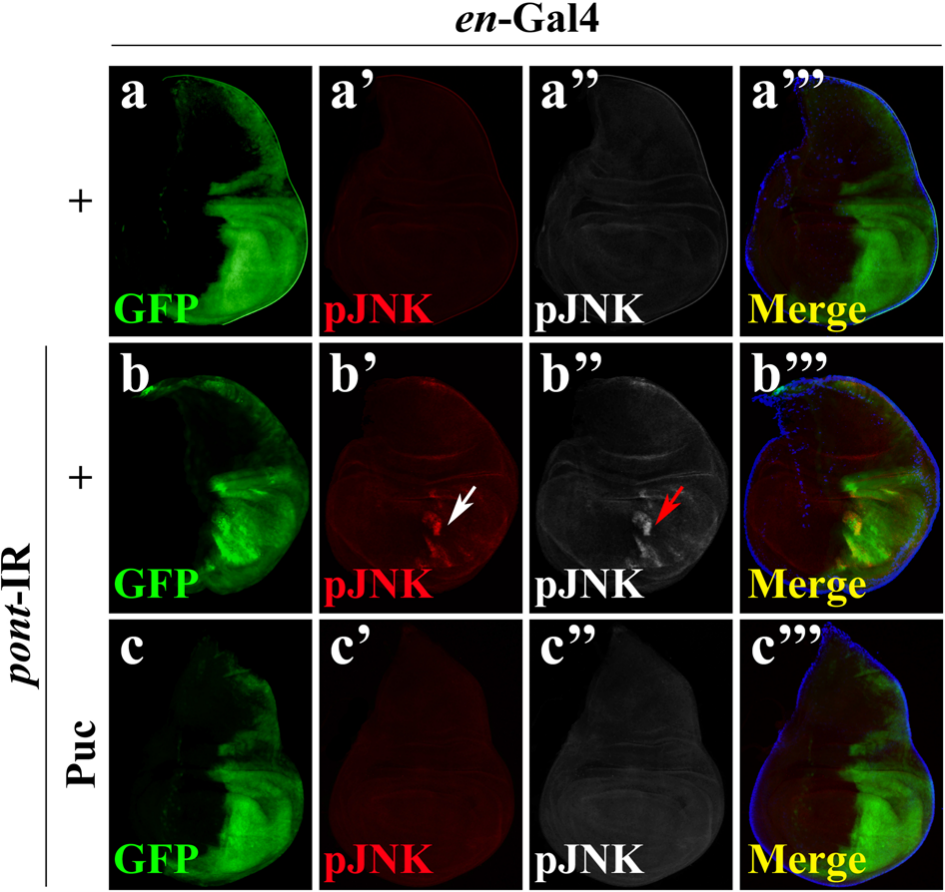
Loss of function of Pont triggers JNK phosphorylation. Fluorescent images of the immunostaining for JNK phosphorylation (pJNK) (**a**–**a”’**, **b**–**b”’**, **c**–**c”’**) are shown. *en*-Gal4 was used as a control (**a**–**a”’**), or to drive the expression of *pont*-IR (**b**–**b”’**, **c**–**c”’**) or *UAS-*Puc (**c**–**c”’**). Compared with the *en*-Gal4 alone (**a**–**a”’**), *en*>*pont*-IR induced strong JNK phosphorylation (**b’**, **b”**, **b”’**, indicated by white arrow in **b’** and red arrow in **b”**), which was abolished by expression of Puc (**c**–**c”’**). Scale bar, 200 μm. Genotypes: *en*-Gal4 *UAS*-GFP/+ (**a**–**a”’**); *en*-Gal4 *UAS*-GFP /*UAS*-*pont*-IR #2 (**b**–**b”’**); *en*-Gal4 *UAS*-GFP /*UAS*-*pont*-IR #2; *UAS*-Puc/+ (**c**–**c”’**)

The above data demonstrate a role of Pont in negatively regulating Egr-JNK signaling. To elucidate how Pont regulates Egr-JNK in development, we checked the immunostaining of phosphorylation of JNK (pJNK), which represents the direct activation of the JNK signaling^40^. Compared with the *en*-Gal4 control (Fig. 4a–a”’), RNAi-mediated downregulation of *pont* in the posterior compartment of the wing disc resulted in strong cell death (Fig. 2b, g) and JNK phosphorylation (Fig. 4b–b”’), which was suppressed by expression of JNK phosphatase Puc (Figs. 2c–g and 4c–c”’). Collectively, the data indicate Pont is a negative regulator of JNK phosphorylation during development.

### Expression of Pont inhibits Egr-induced cell death

**Fig. 5:**
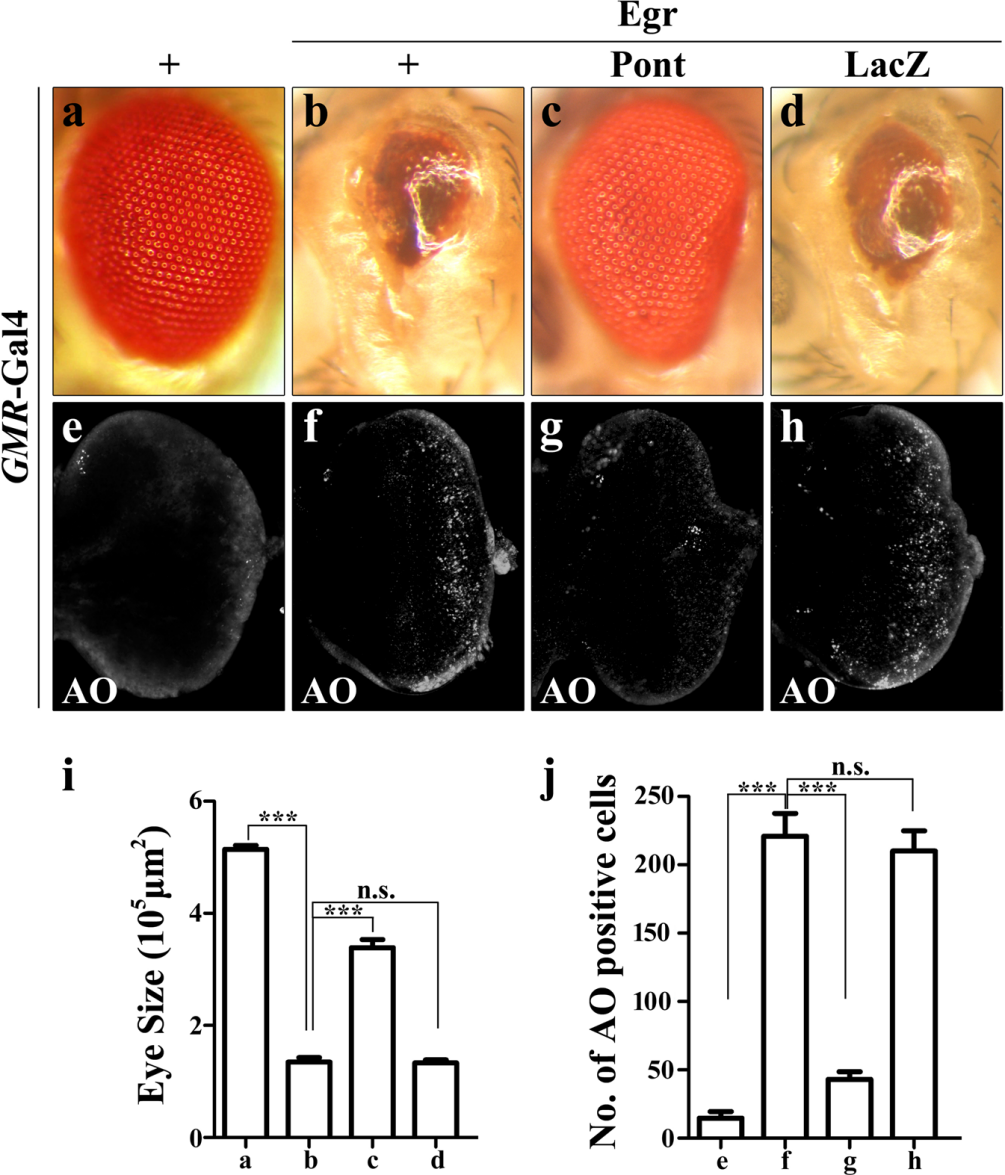
Expression of Pont inhibits Egr-induced cell death in the developing eye. Light images of adult eyes (**a**–**d**) and fluorescent images of acridine orange staining (**e**–**h**) are shown. *GMR*-Gal4 was used as a control (**a**, **e**) or to drive the expression of Egr (**b**–**d**, **f**–**h**) or *UAS-*Pont (**c**, **g**), *UAS-*LacZ (**d**, **h**). *GMR*>Egr-induced small eye phenotype (**b**) and cell death (**f**) were suppressed by expression of Pont (**c**, **g**) not by expression of LacZ (**d**, **h**). **i** Statistical analysis of the eye size in (**a**–**d**). Error bars means ± SEM, ***P ≤ 0.001, n.s. not significant. **j** Statistical analysis of acridine orange-positive cells in (**e**–**h**). Error bars means ± SEM, ***P ≤ 0.001, n.s. not significant. Scale bar, 100 μm. Genotypes: *GMR*-Gal4/+ (**a**, **e**); *UAS-*Egr^S^/+; GMR-Gal4/+ (**b**, **f**); *UAS-*Egr^S^/*UAS*-Pont*; GMR*-Gal4/ *UAS*-Pont (**c**, **g**); *UAS-*Egr^S^/+; *GMR*-Gal4/ *UAS*-LacZ (**d**, **h**)

The above data argue a role of Pont in regulating Egr-JNK-induced cell death and JNK activation. Next we checked the overexpression of Pont to see if gain of Pont could suppress Egr-induced cell death phenotype. Targeted expression of a strong form of Egr in the eye disc driven by *GMR* promoter induced strong cell death (Fig. 5f–j) and produced a small eye phenotype with reduced eye tissues (Fig. 5a, b, i). Consistent with our hypothesis, ectopic expression of Pont (Fig. S2) showed a strong suppression effect on the Egr-induced small eye phenotype (Fig. 5c–i) and cell death in the eye disc (Fig. 5g–j), while no effect was observed in control groups (Fig. 5d, h, i, j), indicating Pont is a negative regulator of Egr in the developing eye.

**Fig. 6:**
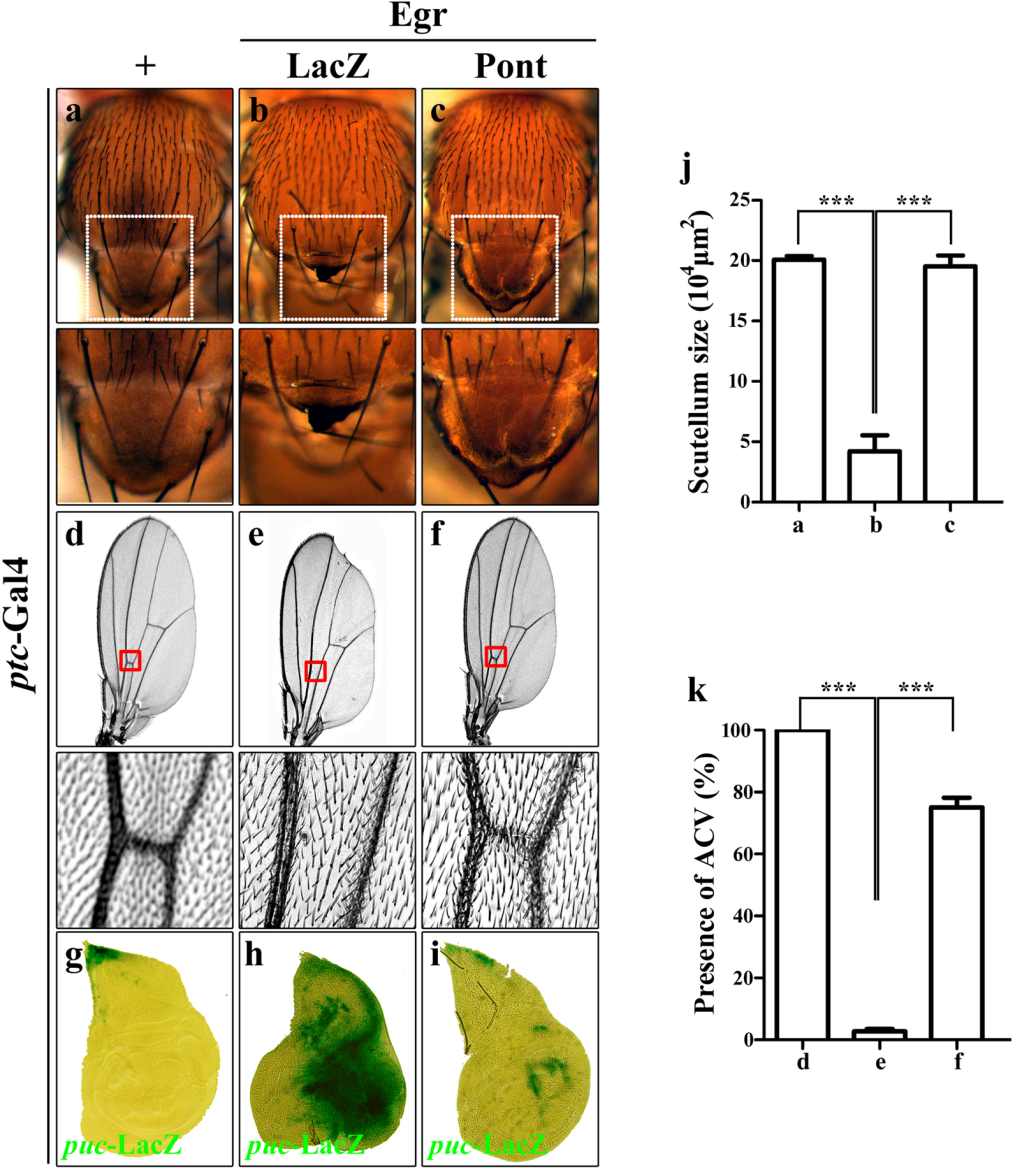
Expression of Pont inhibits Egr-induced cell death phenotype in the thorax and wing. Light images of *Drosophila* adult thoraxes (**a**–**c**), *Drosophila* wings (**d**–**f**) and X-gal staining of third-instar larva wing disc (**g**–**i**) are shown. *ptc*-Gal4 was used as a control (**a**, **d**, **g**), or to drive the expression of Egr (**b**, **c**, **e**, **f**, **h**, **i**) or *UAS-*Pont (**c**, **f**, **i**), or *UAS-*LacZ (**b**, **e**). The lower panels are magnification of the boxed area in the upper panels. Compared with the *ptc*-Gal4 control (**a**, **d**, **g**), *ptc*>Egr-induced small scutellum (**b**), loss of anterior cross vein (ACV) phenotype (**e**) and *puc* activation (**h**) were suppressed by expression of Pont (**c**, **f**, **i**). **j** Statistical analysis of the scutellum size in (**a**–**c**). Error bars means ± SEM, ***P ≤ 0.001. **k** Statistical analysis of the presence of ACV in (**d**–**f**). Error bars means ± SEM, ***P ≤ 0.001. Genotypes: *ptc*-Gal4/+; *puc*^E69^/+ (**a**, **d, g**); *ptc*-Gal4 *UAS-*Egr^W^/+; *puc*^E69^/*UAS*-LacZ (**b**, **e**); *ptc*-Gal4 *UAS-*Egr^W^/+; *puc*^E69^/+ (**h**); *ptc-Gal4 UAS-*Egr^W^ /*UAS*-Pont*; puc*^E69^/ *UAS*-Pont (**c**, **f**, **i**)

Next we wanted to know if the genetic interaction between Egr and Pont is tissue specific. Egr-induced cell death phenotypes in the thorax and wing were examined. Expression of Egr in the wing disc initiated by *patched*-Gal4 (*ptc*-Gal4)^40,51^, which is expressed along the anterior/posterior (A/P) boundary and shows a strong expression pattern in the wing tips which develop into the notum of the *Drosophila*, induced strong cell death and almost abolished the scutellum (Fig. 6a, b, j). Consistently, ectopic expression of Pont significantly restored *ptc*>Egr-induced small scutellum phenotype to that of the control group (Fig. 6a, b, c, j, lower panels). In the adult wing, *ptc*>Egr produced a loss of anterior cross vein (ACV) and a notching phenotype in the wing margin (Fig. 6d, e, k), which was immensely suppressed by gain of Pont function (Fig. 6f–k). Collectively, the data suggest that Pont is a negative regulator of Egr-induced cell death phenotype in *Drosophila*. To understand how Pont regulates Egr signaling, we checked Egr target gene *puc* expression. Expression of Egr along the A/P boundary induced strong cell death (Fig. 6b, e, j, k, data not shown) and *puc* activation (Fig. 6g, h). Consistent with the role of Pont in suppressing Egr-induced cell death phenotype in the wing and thorax, ectopic Egr-induced *puc* activation was largely blocked by expression of Pont (Fig. 6h, i). Taken together, our observation suggests that Pont is required for the endogenous Egr-JNK signaling pathway.

### Pont acts downstream of Hep in the Egr-JNK pathway

**Fig. 7:**
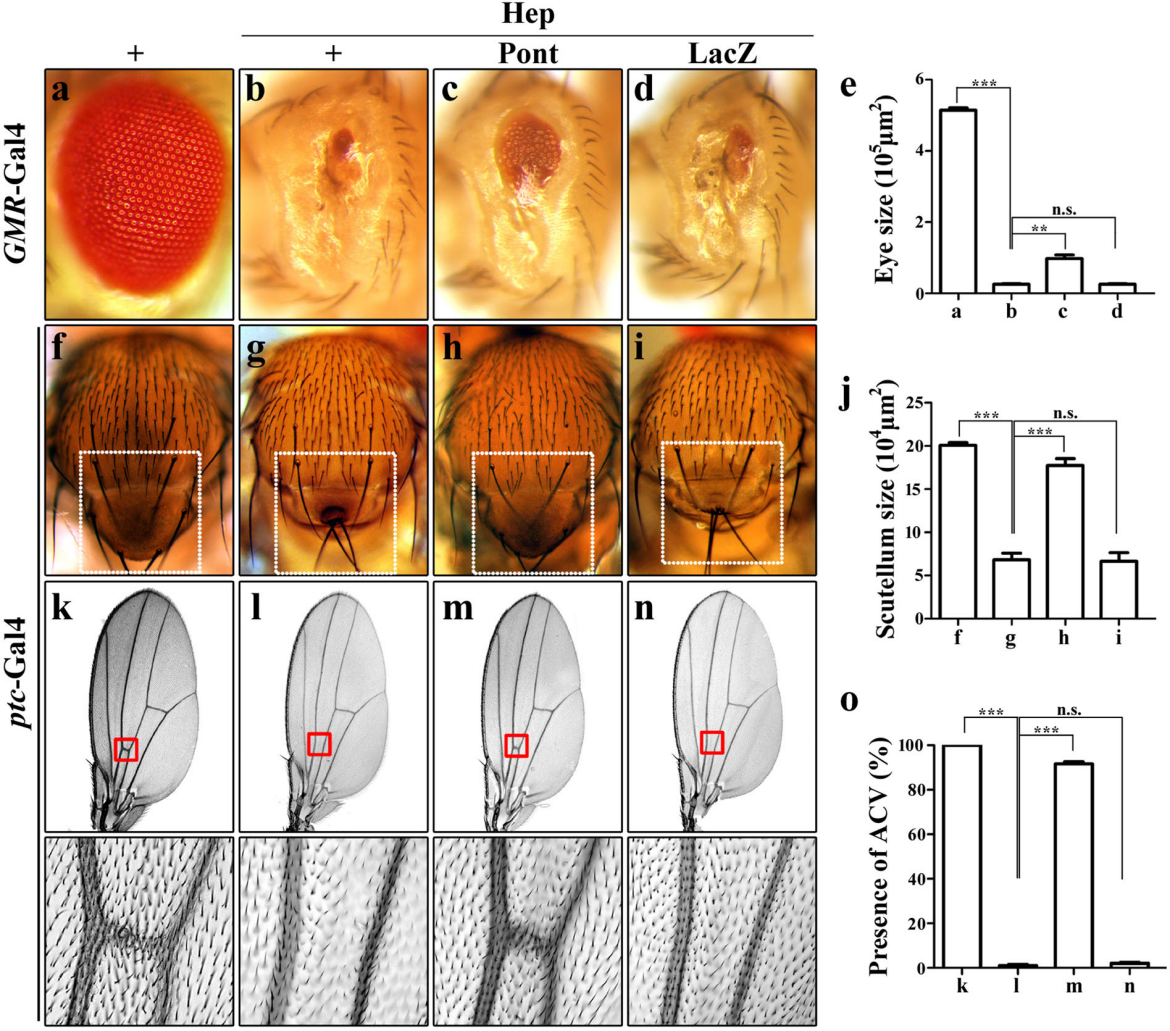
Expression of Pont inhibits Hep-induced cell death. Light images of *Drosophila* adult eyes (**a**–**d**), thoraxes (**f**–**i**) and *Drosophila* wings (**k**–**n**) are shown. *GMR*-Gal4 was used as a control (**a**) or to drive the expression of Hep (**b**–**d**) or *UAS-*Pont (**c**) or *UAS-*LacZ (**d**). *GMR*>Hep-induced cell death phenotype (**b**) was suppressed by expression of Pont (**c**) and not by expression of LacZ (**d**). *ptc*-Gal4 was used as a control (**f**, **k**) or to drive the expression of Hep (**g**–**i**, **l**–**n**) or *UAS-*Pont (**h**, **m**) or *UAS-*LacZ (**i**, **n**). *ptc*>Hep-induced small scutellum phenotype (**g**) and loss of ACV phenotype (**l**) were suppressed by expression of Pont (**h**, **m**) and not by expression of LacZ (**i**, **n**). The lower panels are the magnification of boxed area in the upper panels. **e** Statistical analysis of the eye size in (**a**–**d**). Error bars means ± SEM, ***P ≤ 0.001, **P ≤ 0.01, n.s. not significant. **j** Statistical analysis of the scutellum size in (**f**–**i**). Error bars means ± SEM, ***P ≤ 0.001, n.s. not significant. **o** Statistical analysis of the presence of ACV in (**k**–**n**). Error bars means ± SEM, ***P ≤ 0.001, n.s. not significant. Genotypes: *GMR*-Gal4/+ (**a**); *GMR*-Gal4 *UAS*-Hep^CA^/+ (**b**); *UAS*-Pont/+; *GMR*-Gal4 *UAS*-Hep^CA^/*UAS*-Pont (**c**); *GMR*-Gal4 *UAS*-Hep^CA^/*UAS*-LacZ (**d**); *ptc*-Gal4/+; *puc*^E69^/+ (**f**, **k**); *ptc*-Gal4/*UAS*-Hep^WT^; *puc*^E69^/+ (**g**, **l**); *ptc*-Gal4 *UAS*-Hep^WT^/*UAS*-Pont; *UAS*-Pont/ *puc*^E69^ (**h**, **m**); *ptc*-Gal4 *UAS*-Hep^WT^/+; *UAS*-LacZ/ *puc*^E69^ (**i**, **n**)

To genetically locate the epistasis of Pont in the Egr-JNK pathway, we examined the genetic interaction between Hep (JNKK) and Pont in the developing eye, thorax and wing. Expression of a constitutive active form of Hep in the developing eye (*GMR*>Hep^CA^) triggers JNK-mediated cell death and gives rise to small eyes with a reduced photoreceptor cells phenotype (Fig. 7a, b, e)^40^. Expression of Pont compromised *GMR*>Hep^CA^-induced phenotype (Fig. 7c–e), while expression of randomly inserted LacZ transgene showed no effect (Fig. 7d, e). Similar to ectopic Egr (*ptc*>Egr) producing a small scutellum and loss of ACV phenotype under the control of *ptc*-promoter, the expression of wild type of Hep (*ptc*>Hep) also generates such phenotype (Fig. 7f, g, j, k, l, o). Consistent with the observation in the eye, expression of Pont but not LacZ shows a strong suppression effect of *ptc*>Hep-induced small scutellum and loss of ACV phenotype (Fig. 7h–j, m–o). Combining the above data, we conclude that Pont modulates Egr-JNK pathway at downstream of Hep through the phosphorylation of Bsk.

## Conclusion

In the present work, we have identified ATPase Pontin as a crucial modulator of the conserved Egr-JNK signaling during the development of *Drosophila*. Our genetic evidence revealed that Pontin is a negative regulator of Egr-JNK cascade. We showed that loss of function of Pontin promoted while gain of function of Pontin suppressed Egr-induced cell death. Consistent with our observation, dominant-negative mutant of Pontin potentiates the apoptotic activity of c-Myc and E2F1^52^ and in HCC cells the knockdown of Pont also led to spontaneous apoptosis^53^. We further showed that Pontin acted downstream of Hep in the Egr-JNK pathway to induce JNK-mediated *puc* activation and scutellum development. Finally, we demonstrated that Pontin was a negative regulator of JNK phosphorylation, for loss of function of Pontin was sufficient to induce JNK phosphorylation in vivo. The work proposes a novel role of ATPase in the modulating of JNK pathway in vivo and further study should clarify if other ATPases also interact with the JNK cascade.

## Materials and methods

### Fly stocks

All stocks were raised on standard *Drosophila* media and crosses were performed at 25 °C unless otherwise indicated. *UAS*-*pont*-IR #1 and *UAS*-*pont*-IR #2 were obtained from NIG stock center. *bsk*^1/+^, *bsk*^2/+^, *fos*^1/+^, *UAS*-LacZ, *UAS*-*GFP*-IR and *en*-Gal4 were obtained from the Bloomington *Drosophila* Stock Center. *UAS*-Pont, *pont*^5.1^/TM3, Ser^11^, *puc*^E69^, *UAS*-Egr^w^, *UAS*-Egr^R^ ^17^, *UAS*-Hep^WT^ ^40^, *UAS*-Hep^CA^ ^42^, *UAS*-Puc^40^, *GMR*-Gal4, *ptc*-Gal4, *sd*-Gal4, *pnr*-Gal4 and *ap*-Gal4^51^ have been previously described. The third-instar larvae were heated-shocked at 37 °C for 1 h and allowed to recover for 2 h at 25 °C.

### Light image

Flies of indicated genotypes were collected and immediately frozen in −80 °C. Flies were placed on the 1% agarose plate before images taking. Wings were dissected and mounted on the slide in the alcohol/glycerol (1:1) medium and flies were mounted on the 1% agarose plate in the alcohol/glycerol medium for the image taking of thoraxes. Light images of wings were collected with Olympus microscope BX51, and light images of thoraxes were collected with OLYMPUS stereo microscope SZX16.

### AO staining

Wing discs were dissected from the third-instar larvae in 1% phosphate-buffered saline (PBS) buffer and stained for acridine orange as previously described^40^. Each genotype was dissected with 20 discs for statistics.

### X-gal staining

Wing discs were dissected from the third-instar larvae in 1% PBS buffer and stained for β-galactosidase (β-gal) activity as previously described^54^.

### Immunohistochemistry

The third-instar larvae of indicated genotypes were collected and dissected in 1% PBS buffer. The antibody staining of imaginal discs was conducted as previously described^18^. The following antibodies were used: mouse anti-β-gal (1:400, Developmental Studies Hybridoma Bank), rabbit anti-phospho-JNK (1:200, Calbiochem) and rabbit anti-cleaved caspase-3 (1:400, Cell Signaling and Technology); secondary antibodies were anti-rabbit-Alexa (1:1000, Cell Signaling and Technology) and anti-mouse-Cy3 (1:1000, Jackson ImmunoResearch).

## Electronic supplementary material

Figure S1

Figure S2

Figure S3

Figure S4

Figure S5

Supplementary figure legends

## Notes

**Supplementary Information** accompanies this papaer at 10.1038/s41420-018-0074-1.
