# Supplementary figures and images for: Pontin/Tip49 negatively regulates JNK-mediated cell death in Drosophila
Source: Cell Death Discov. 2018 Jul 9;4:74. doi: 10.1038/s41420-018-0074-1 (PMC6060144; doi:10.1038/s41420-018-0074-1)

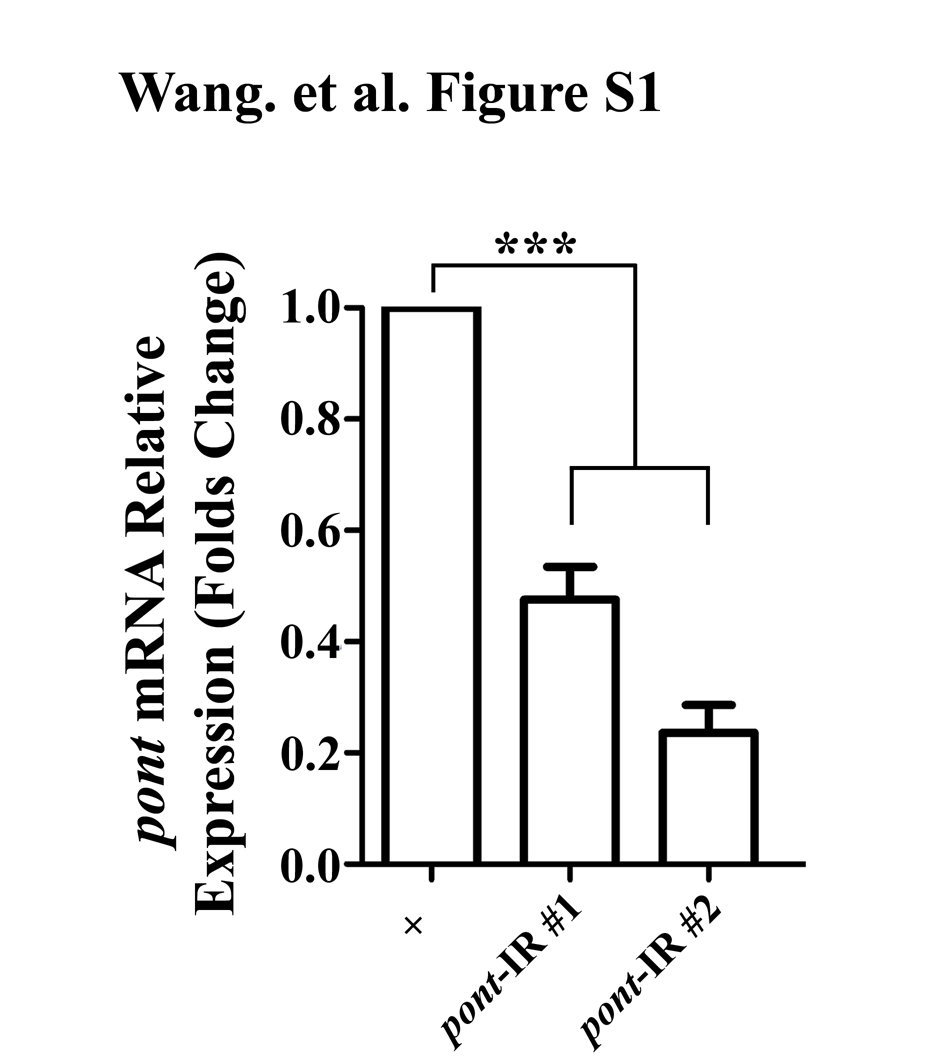

Supplement: Supplementary file 1 — Figure S1 [file 41420_2018_74_MOESM1_ESM.jpg]

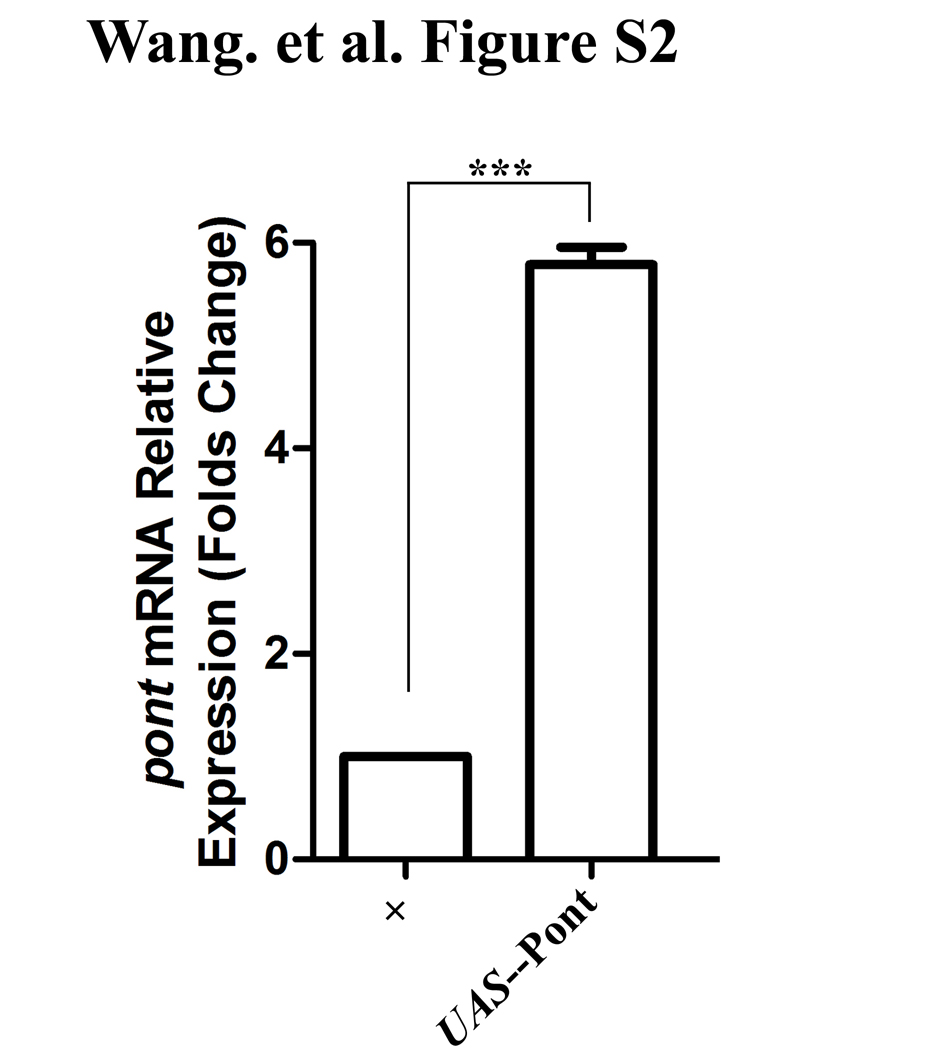

Supplement: Supplementary file 2 — Figure S2 [file 41420_2018_74_MOESM2_ESM.jpg]

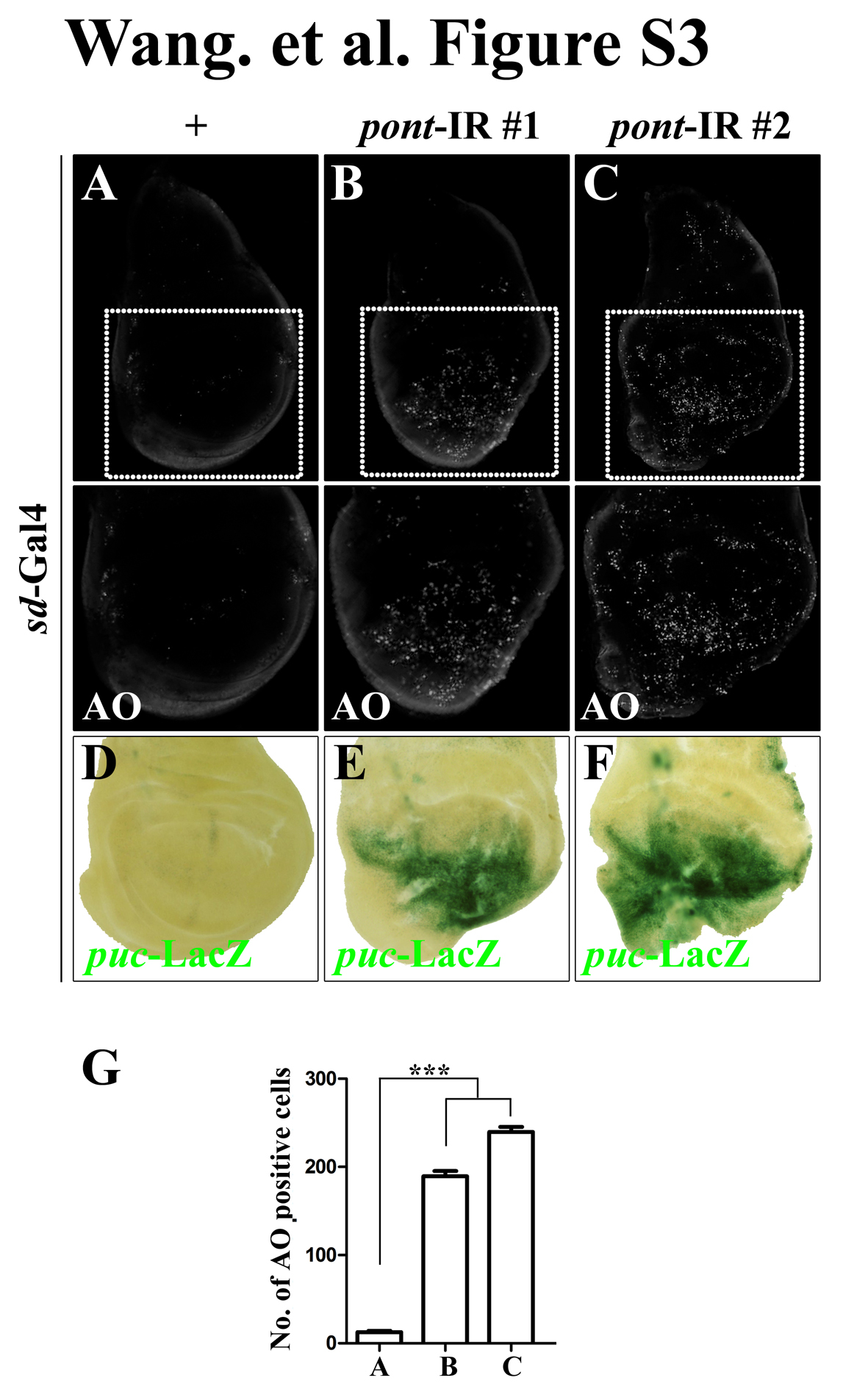

Supplement: Supplementary file 3 — Figure S3 [file 41420_2018_74_MOESM3_ESM.jpg]

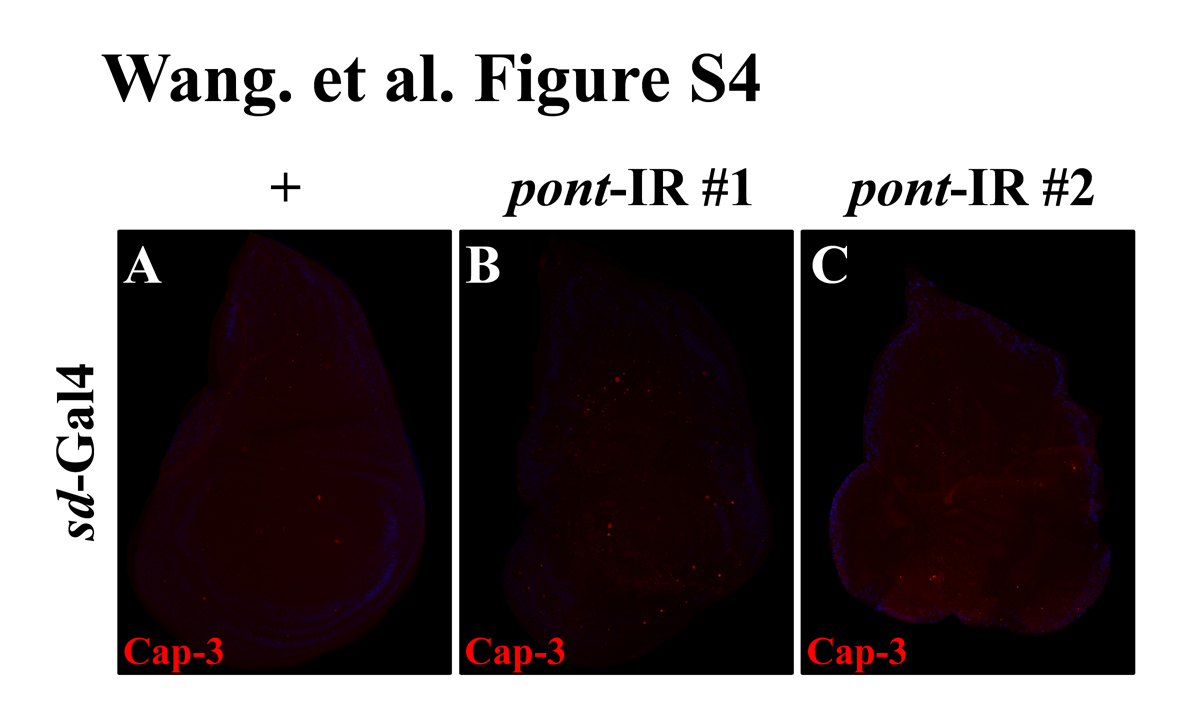

Supplement: Supplementary file 4 — Figure S4 [file 41420_2018_74_MOESM4_ESM.jpg]

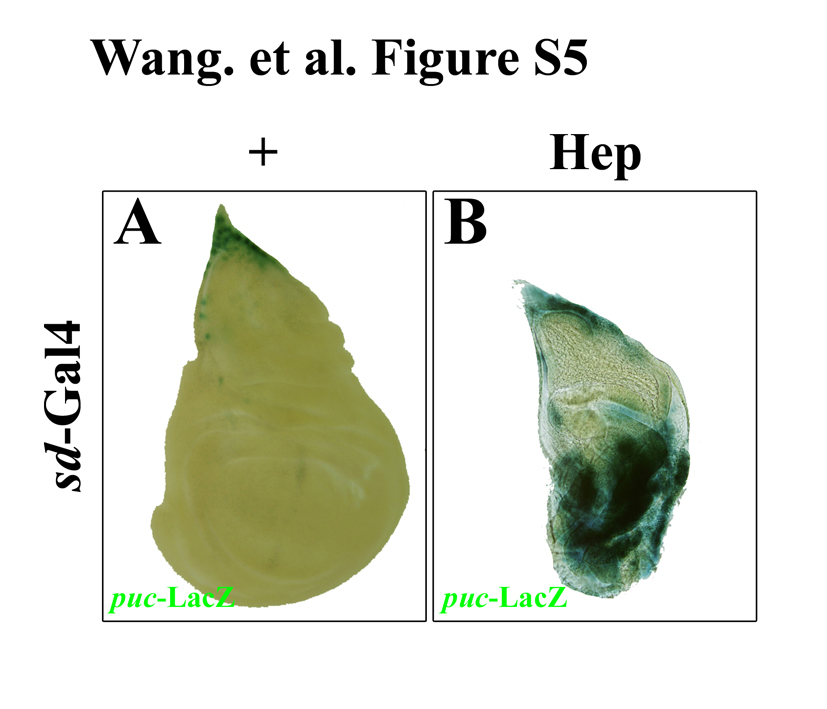

Supplement: Supplementary file 5 — Figure S5 [file 41420_2018_74_MOESM5_ESM.jpg]
