# Supplementary material for: Pontin/Tip49 negatively regulates JNK-mediated cell death in Drosophila
Source: Cell Death Discov. 2018 Jul 9;4:74. doi: 10.1038/s41420-018-0074-1 (PMC6060144; doi:10.1038/s41420-018-0074-1)
Supplement: Supplementary file 6 — Supplementary figure legends [file 41420_2018_74_MOESM6_ESM.docx]

**Supporting figures**

**Figure S1. Quantitative analysis of *pont* mRNA expression**

RT-PCR analysis of *pont* mRNA expression in 3^rd^ instar larva was shown. The 3^rd^ instar larva were heat shocked in 37℃ for 1 hour and recovered for 2 hours in 25℃. Error bars means ±SEM, ***: P≤ 0.001.

**Figure S2. Quantitative analysis *of* mRNA expression of *ectopic pont***

RT-PCR analysis of *pont* mRNA expression in 3^rd^ instar larva was shown. The 3^rd^ instar larva were heat shocked in 37℃ for 1 hour and recovered for 2 hours in 25℃. Error bars means ±SEM, ***: P≤ 0.001.

**Figure S3. Loss-of-function of Pont triggers JNK-mediated cell death in larval wing discs**

Compared with *sd*-Gal4 alone (**a**, **d**), expression of *pont*-IR in the wing pouch driven by *sd*-Gal4 induced strong cell death (**b**, **c**) with elevated *puc* activation (**e**, **f**). The lower panels are the magnification of the boxed area in the upper panels. **g** is statistical analysis of acridine orange-positive cells in **a**-**c**. Error bars means ±SEM，***: P≤ 0.001. Scale bar for **a-c**, 200 μm.

**Genotypes**: *sd*-Gal4/+ (**a**); *sd*-Gal4/+; *UAS*-*pont*-IR #1/+ (**b**); *sd*-Gal4/+; *UAS*-*pont*-IR #2/+ (**c**); *sd*-Gal4/+; *puc*^E69^/+ (**d**); *sd*-Gal4/+; *puc*^E69^/ *UAS*-*pont*-IR #1 (**e**); *sd*-Gal4/+; *UAS*-*pont*-IR #2/+; *puc*^E69^/+ (**f**);

**Figure S4. Loss-of-function of Pont induces Caspase-independent cell death in larval wing discs**

Fluorescent images of the immunostaining cleaved caspase 3 (**a-c**) are shown. *sd*-Gal4 was used as a control (**a**), or to drive the expression of *pont*-IR (**b**, **c**). Compared with the *sd*-Gal4 alone (**a**), *sd*> *pont*-IR failed to induce evident cleaved caspase 3 activation in the wing disc (**b**, **c**). Scale bar for **a-c**, 200 μm.

**Genotypes**: *sd*-Gal4/+ (**a**); *sd*-Gal4/+; *UAS*-*pont*-IR #1/+ (**b**); *sd*-Gal4/+; *UAS*-*pont*-IR #2/+ (**c**).

**Figure S5. Expression of Hep induces *puc* transcription in the wing disc**

Light images of X-gal staining of 3rd instar larva wing disc (**a**, **b**) are shown. sd-Gal4 (**a**) was used as a control or to drive the expression of Hep (**b**). Compared with the sd-Gal4 alone, expression of Hep strong activated puc transcription in the wing disc (**b**).

Genotypes: s*d*-Gal4/+; *puc*^E69^/+ (**a**); *sd*-Gal4/+; *UAS*-Hep^WT^/+; *puc*^E69^/+ (**b**).
